# Research Progress and Hopeful Strategies of Application of Quorum Sensing in Food, Agriculture and Nanomedicine

**DOI:** 10.3390/microorganisms10061192

**Published:** 2022-06-10

**Authors:** Gennaro Roberto Abbamondi, Giuseppina Tommonaro

**Affiliations:** Institute of Biomolecular Chemistry (ICB), National Research Council of Italy (CNR), Via Campi Flegrei 34, 80078 Pozzuoli, NA, Italy; giuseppina.tommonaro@icb.cnr.it

**Keywords:** quorum sensing (QS), quorum quenching (QQ), QS inhibition (QSI), food, agriculture, medicine, nanotechnology, application of QS

## Abstract

Quorum sensing (QS) regulates the expression of several genes including motility, biofilm development, virulence expression, population density detection and plasmid conjugation. It is based on “autoinducers”, small molecules that microorganisms produce and release in the extracellular milieu. The biochemistry of quorum sensing is widely discussed and numerous papers are available to scientists. The main purpose of this research is to understand how knowledge about this mechanism can be exploited for the benefit of humans and the environment. Here, we report the most promising studies on QS and their resulting applications in different fields of global interest: food, agriculture and nanomedicine.

## 1. Introduction

Microbial populations inhabit terrestrial and aquatic microenvironments, with a great variety of ecological systems [1]. In order to successfully colonize an ecological niche, microorganisms develop a great capability to adapt to multiple environmental parameters, such as temperature, pH, salt concentration and hydrostatic pressure. Moreover, their establishment and persistence in a specific ecosystem is strictly correlated to competition dynamics and ecological interactions. Intra- and inter-species microbial interactions and even microorganism–host interactions have a key role in preserving species diversity of specific microenvironments, which is correlated to the increasing of the competitive ability of the community [2]. These complex network systems can be beneficial (mutualism, commensalism), neutral (neutralism) or disadvantageous (amensalism, parasitism, competition) for the individual microorganism [3,4]. Recent development of multi-omic approaches have given a strong boost to the understanding of bacterial communities, but the prediction of their complex relationships is still difficult to interpret [5].

The idea that bacteria were autonomous unicellular organisms without capacity of collective behavior has been abandoned. Nowadays, it is generally accepted that microorganisms are able to communicate with each other by means of a molecular language. A density-dependent cell-signaling system has been described as quorum sensing by Fuqua in 1994 [6], and pioneered the concept of social microbiology [7]. Quorum sensing (QS) is based on small signal molecules, named autoinducers (AI), which bacteria release in the extracellular milieu. These molecules are sensed by the bacterial community (links to receptor protein) and activate the coordinated gene expression only when the cells reach a quorum [8]. This concentration-dependent transcriptional regulation is associated with various phenotypes and physiological activities, including motility, biofilm development, virulence expression, population density detection and plasmid conjugation [9,10].

## 2. Quorum Sensing and Autoinducers

Different classes of signal/receptor couples have been described, which regulate different QS systems. The most studied signal molecules are N-Acyl-Homoserine Lactones (AHLs), used by many Gram-negative bacteria and involved in the QS AI-1 system [11]. The AI-1 mechanism is based on AHL synthetases (LuxI or LuxM) which produce the AHL from S-adenosylmethionine (SAM); the chemical signal is released in the extracellular milieu and, when a certain concentration is reached, it binds to intracellular luxR-type receptors which mediate a concentration-dependent transcriptional regulation.

Gram-positive bacteria mainly communicate with each other by synthesizing modified oligopeptides (or autoinducer peptides—AIP) [12]. These short peptide chains are synthesized by ribosomes as pro-peptides and post-translationally modified. Two major QS pathways have been described in Gram-positive bacteria. AIPs are secreted in the extracellular milieu where they reach a threshold concentration. Then, in the “Self-Signaling Pathway” they are reinternalized via an oligopeptide transporter system; differently, in the “Two Component Pathway”, they bind and activate a receptor His kinase on the cell membrane, which eventually activates an intracellular regulator via phosphor transfer, inducing an increased expression of the target gene [13].

**Figure 1:**
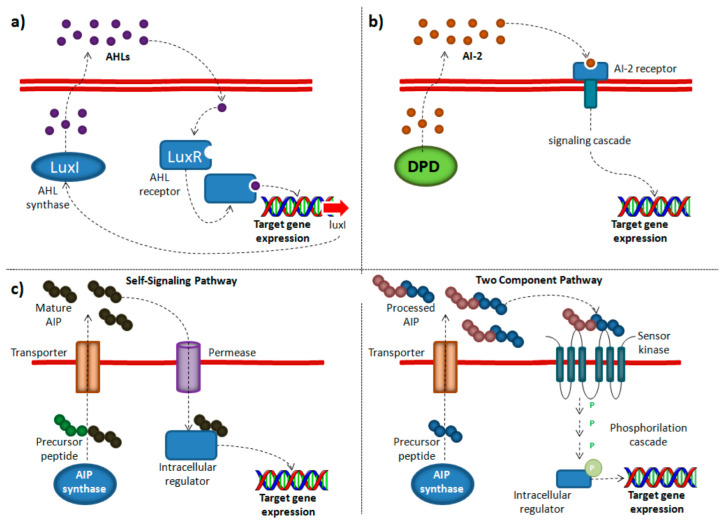
Simplified QS circuit diagrams of (**a**) AI-1, (**b**) AI-2 and (**c**) AIP (“Self-Signaling” and “Two Component” pathways) mechanisms.

Small cyclic furanone compounds (AI-2 system) participate in signal transduction in different bacterial species; because they are widespread among Gram-positive and Gram-negative bacteria, AI-2 has been proposed as a “universal signaling system”, but this role is still debated [14]. Autoinducer-2 is a byproduct of the activated methyl cycle, with 4,5 dihydroxy-2,3-pentanedione (DPD) as a precursor that can rearrange to R- or S-2-methyl-2,3,3,4-tetrahydroxytetrahydrofuran, which can form borate complexes in the presence of environmental boron [15]. Figure 1 summarizes the most common QS mechanisms.

Since the study of QS is expanding, attracting growing interest of researchers, novel QS signals have been discovered. Recently, the structural, biochemical and functional characterization of a third autoinducer system, AI-3, has been identified, and 3,6-dimethylpyrazin-2-one was designated as the involved signal molecule [16,17].

Cis-2-unsaturated fatty acids have been reported as a QS signal in different Gram-negative bacteria pathogens and have been described as a diffusible signal factor (DSF) [18,19].

From diverse species of *Pseudomonas* and *Burkholderia,* 4-Hydroxy-2-alkylquinolines (HAQs) have been isolated which have been reported as an additional QS molecule class [20].

The presence of QS has been observed among eukaryotes as well. Specifically, it was first observed in the fungal pathogen *Candida albicans*, in which farnesol was indicated as responsible for QS activity [21]. Since then, several autoinducers have been described in fungi, mainly aromatic alcohols derived from the amino acids tyrosine (tyrosol), phenylalanine (2-phenylethanol) and tryptophan (tryptophol), but also lipids (oxylipins) and peptides (pheromones) [22,23].

In *Vibrio cholerae*, virulence gene expression is regulated by the concerted action of AI-2 and *V. cholerae* autoinducer-1 (CAI-1). In particular, CAI-1 ((S)-3-hydroxytridecan-4-one) is synthesized by the CqsA and released out of the cell; when the threshold concentration is reached, it binds the membrane protein CqsS which is phosphorylated and activates LuxO (via LuxU) [24].

Even if further research is needed, the detection of signal molecules, mainly AHLs, in the extracellular media of extremophilic microorganisms (which thrive in harsh environments), demonstrates the ubiquity of QS systems, making research on molecular communication even more intriguing and with multidisciplinary interests [25].

## 3. Quorum Sensing in Agriculture and Food

The constant growth of the world population and the consequent need to produce a sufficient quantity of food has become a central topic of political and public debate. In fact, in the last 20 years, with an even higher rate since 2009, several projection studies on food safety have been published. The analysis of this research showed that between 2010 and 2050, food demand is expected to grow between +35% and +56%, with a consequent risk of rising global hunger [26]. In this scenario, the development of sustainable food production strategies becomes a primary challenge, in order to increase both production and quality of food items, limiting harmful environmental effects. In fact, intensive agricultural production is currently dependent on the use of chemical fertilizers and manures, as well as pesticides, with known negative effects on the environment (leaching of nitrate into ground water, phosphorus and nitrogen run-off, aquatic ecosystem eutrophication).

To fill the growing need for food, the synergistic action of several strategies is necessary: increase in production yield, reduction in food spoiling, and gain in food quality.

As far as agriculture is concerned, a higher yield can be achieved, in a sustainable way, through technological systems based on the inoculation of selected microorganisms [27]. We refer to plant growth promotion and plant disease control as innovative techniques which modulate the microbial population associated with the plant to favor its growth and its resistance to adverse situations (diseases, pathogens, drought, etc.) [28]. It is clear that multicellular organisms collectively form a holobiont with their microbiota, which deeply contributes to their physiology and development [29]. The network between the plant host and the related microbial community is strictly correlated to quorum-sensing signaling systems, and it is of primary importance for the constitution of the holobiont. Interkingdom signaling affects the balance of pathogenic or beneficial bacteria and their host plants, influencing plant growth and immunity. It has been reported that bacterial AHL signals influence plant performance, even if the specific molecular mechanisms of their action on the plants needs further study to be fully elucidated [30]. Several studies investigated the effects of AHL signals on the most popular model plant *Arabidopsis thaliana*. Depending on the molecular structure of the QS signal, it has been demonstrated that AHLs induce changes in the phytohormone balance, mediate morphological changes in roots (stimulation of root growth, and primary root elongation), enhance tolerance to salt stress, etc. [31]. Moreover, the presence of luxR-solo or luxR-orphan genes were found in different plant-associated bacteria; it is a special condition in which the microbial chromosome harbors the luxR gene, while the gene encoding the corresponding luxI-type AHL synthase is missing. These so-called solo luxR genes may respond to exogenous AHL or even signals from plants, which open a new perspective on the understanding of interkingdom interactions [32].

Furthermore, it is reported that QS is involved in the optimization of virulence also in plant pathogenic bacteria; therefore, interference in QS can lead to a reduction in their pathogenicity. Specifically, quorum quenching (QQ) is the term adopted to describe the disruption of quorum sensing. Quenching certain pathogen signals can be considered a promising strategy to counteract plant infections [33].

On the other hand, a reduction in food waste can be achieved by reducing its spoilage. The finding of innovative and sustainable strategies against food deterioration is attracting more and more attention in the world of research. The spoilage is mainly correlated with the enzymatic activity of microorganisms that, finding a favorable environment, grow inside the food and cause varying degrees of change in its characteristics. Food should in fact be analyzed as an ecosystem, in which the microbial network plays a pivotal role in the edible product quality. Foodborne pathogens and food spoilage organisms represent a problem of global concern. Microbial toxins contaminate any type of food and water, endangering public health conditions. The classical examples of food and waterborne pathogens are *Staphylococcus aureus*, which produces heat-stable enterotoxins, causing gastrointestinal symptoms, and *Salmonella Typhimurium,* which causes Typhoid fever and salmonellosis by synthesized enterotoxin. Takó et al. recently described the most common foodborne pathogenic microorganisms, linking them with the main contaminated food sources, their produced toxins and their effect on human health [34]. The metabolic end products of proteolytic, lipolytic, pectinolytic and saccharolytic activity can be correlated with food spoiling. Several of those enzymes are under QS control; therefore, a better understanding of communication mechanisms in food ecosystems can contribute to reducing food waste, limiting its deterioration [35]. Moreover, foodborne pathogens can easily form biofilms on a wide variety of abiotic or biotic surfaces, such as plastic, glass, metal and wood, which are generally used as packaging by the food industry. This specific lifestyle of microorganisms, which are embedded in a self-produced extracellular polymeric matrix, improves their capability to survive under adverse environmental factors, decreasing their susceptibility to antibiotics, making them more difficult to eliminate by mean standard cleaning and disinfection procedures [36]. Biofilm development, mainly in the sessile growth phase, is regulated by intracellular QS interaction [37]. This happens also in different pathogens, including, for example, *Pseudomonas aeruginosa*, which can colonize and then be transmitted also by food, establishing opportunistic infections with high mortality rates [38]. The potential role of QS in spoilage has recently attracted growing research interest. QS signal molecules, mainly belonging to AI-1 and AI-2 systems, have been detected in food sources (such as meat, meat products and vegetables) [39]. In particular, most studies mainly point to the exploitation of QS inhibitors (QSI) in the fight against food spoilage microorganisms and foodborne pathogens as a promising strategy in controlling bacterial biofilm formation [40]. Several molecules and extracts from natural sources (plants, bee products, bacteria, algae), such as phenolic compounds and flavonoids, have demonstrated quorum-sensing inhibition against microorganisms which cause food spoilage, with a consequent potential use in the food industry to disrupt the biofilm formation or eliminate already preformed ones. Natural products are preferred by consumers compared to chemically synthesized preservatives, which make them increasingly recognized as relevant for food companies; therefore, an intensification of the efforts in the research for natural QSI is needed [41].

The above-described strategies to counteract the food crisis in an eco-friendly manner are summarized in Table 1.

Moreover, standard laboratory settings are ideal and highly controlled, but cannot extensively represent realistic environments, in which chemical and physical conditions dynamically change. Consequently, further efforts are needed in the study of QS in microbial mixtures of species that mimic real habitat parameters [42].

## 4. Quorum Sensing and Nanomedicine

The use of nanotechnology to prevent and treat human diseases has remarkably developed since the 1990s. Currently, traditional molecular drugs prevail in drug design and development research; however, this research is often accompanied by the use of nanotechnology with the aim to improve the efficiency and to decrease side effects. Indeed, nanotherapeutics frequently display improved effect compared to traditional drugs because of their features (size of 10–100 nm; large surface-area-to-volume ratio; flexibility of surface functionalization and extensive reactivity). These features provide nanotherapeutics an enhanced bioavailability, low toxicity, better pharmacokinetics and therapeutic efficacy. Moreover, the effective delivery and release of drugs to a target is still the main challenge to enhance available therapies for several human diseases. The use of NPs as “transporters” represents a promising strategy for enhancing delivery, targeting and protection of drugs [43,44]. Nanotechnology is a hopeful research area for the treatment and management of bacterial infections, in particular against multidrug-resistant strains and bacterial biofilms. The QS mechanism plays a key role in biofilm formation by pathogenic microorganisms; therefore, innovative therapeutic approaches based on the disruption of microbial QS signaling (QQ) can be effective in the prevention of biofilm-associated infections [45,46]. Several natural compounds, mainly terpenoids (eugenol, carvacrol, phytol, linalool, D-limonene and α-pinene), phenolic acids (salicylic acid, rosmarinic acid, cinnamic acid, chlorogenic acid, *p*-coumaric acid and caffeic acid), flavonoids (epigallocatechin, naringenin, quercetin, naringin, quercetin 4′-O-β-D-glucopyranoside, taxifolin and morin) from plants, as well as enzymes (mainly lactonases, acylases and oxidoreductases) and antibodies (monoclonal antibodies, mAbs RS2-1G9, able to inhibit 3-oxo-C12-AHL-based QS signaling in *P. aeruginosa*), are reported as QQ agents. They display inhibitory activities through different mechanisms and may act on the synthesis of autoinducers (by deregulating the QS gene expression) or by blocking the cellular receptor [47,48,49]. The development of nanotechnology in medicine has led researchers to design nanostructured materials (nanoparticles and nanocapsules) able to interfere with QS involved in biofilm production and growth. The advantages of use of nanomaterials are the controlled release, the precision targeting and the ability to preserve the carrier drug from the unfavorable environment.

The abilities of metals and metallic nanoparticles to exert QQ activity have been particularly pointed out [50]. Silver nanoparticles have been reported as an innovative nanomaterial, exhibiting a remarkable QS inhibition activity [51,52,53]. Selenium (SeNPs) and tellurium (TeNPs) nanoparticles were also examined in two bacterial processes mediated by QS: violacein production by *Chromobacterium violaceum* and biofilm formation by *P. aeruginosa*. Both showed an important disruption of the QS signaling system, supporting nanotechnology as a promising strategy to combat against the bacterial resistance related to bacterial biofilm formation [54]. Furthermore, both gold nanoparticles (GNPs) and GNPs functionalized with tobramycin or/and antimicrobial peptide Pediocin AcH and Listeria adhesion protein (LAP) (GNP–Pediocin–LAP) were reported as very effective against biofilm formation [55,56]. Functionalized nanoparticles of gallium and bismuth also displayed a significant activity against bacterial biofilm by acting on the quorum-sensing mechanism of *P. aeruginosa* [57,58]. The aforementioned examples of representative research in this field are summarized in Table 2.

It is noteworthy the use of functionalized chitosan nanomaterials as an attractive strategy against chronic infections by attenuating quorum sensing, and their use for the preparation of medical devices [59]. The biodegradability, nontoxicity and biocompatibility of chitosan make it suitable in medical use for the delivery and controlled drug release. In particular, chitosan nanoparticles (ChNPs), due to their chemical property and biological activity, have been used for drug delivery, mainly as functionalized NPs [60,61]. The QQ compounds could be encapsulated in chitosan nanocapsules and could be delivered in response to electrostatic interaction of nanocapsules with bacteria, resulting in an enhancement in QS inhibition activity [62].

## 5. Perspective

In the last 10 years, studies on quorum sensing have risen exponentially, revealing its complexity and its key role in different microbial behaviors. Meanwhile, the search for new technology has increased due to the need for innovative and sustainable strategies in agriculture, medicine and the environment. In this context, the understanding of how microbes communicate with each other means to know “when, where and how” to act in order to drive their behavior, both to promote beneficial microbial traits (i.e., plant growth promotion) and to prevent those that are dangerous (i.e., biofilm formation). The most recent investigations are mainly addressed towards the search for new strategies to block the QS mechanism, by targeting autoinducers and/or receptors (quorum quenching), resulting in the development of new antibiotic therapies. Further efforts should be conducted in the promotion of beneficial aspects of QS, such as the progress and management of new eco-sustainable strategies in agriculture (PGPB and bioremediation).

## Figures and Tables

**Table 1:** Representative eco-friendly strategies to overcome food crisis, exploiting QS mechanism.

Target	Strategy	Mechanism	Involvement of QS	References
Enhancement in yield and quality of agricultural products.	Development of systems to influence (improve) plant performance based on microbial inoculation (plant growth promotion, plant disease control).	Improvement in plant growth, resistance to parasites, drought and salinity tolerance, yield increase and improvement in the quality of the final product.	Influence of plant growth performance by bacterial AHL signals. Ability of bacteria to respond to plant signals, also via LuxR solo (orphan LuxR). Quorum quenching (QQ) of pathogen signals.	[28,29,30,31,32,33]
Increasing the shelf life of food products.	Development of systems/materials to counteract food spoilage and reduce food waste.	Inhibition of the enzymatic (proteolytic, lipolytic, pectinolytic and saccharolytic activity) activity of foodborne pathogens.	Involvement of QS in the enzyme and biofilm production of several foodborne pathogenic microorganisms. Exploitation of QS inhibitors (QSI) as a promising strategy in the fight against food spoilage microorganisms and foodborne pathogens.	[34,35,36,37,38,39,40,41]

**Table 2:** Representative examples of nanoparticles or drugs functionalized with nanoparticles developed to produce bacterial biofilm inhibition.

Nanomaterial	Bacterial Model	Observed Results	References
Selenium (SeNPs) and tellurium (TeNPs) nanoparticles	*P. aeruginosa* (biofilm formation); *C. violaceum* ATCC 12472 and CV026 (violacein production).	Biovolume reduction in biofilm developed by *P. aeruginosa*. Inhibition in the violacein production by *C. violaceum*. Putative disturbance of the AI biosynthesis (SeNPs) and QS signal perception and response (TeNPs).	[54]
GNPs functionalized with tobramycin	*P. aeruginosa* (biofilm formation)	Not a suitable nanocarrier due to the premature release of tobramycin from the liposomes upon functionalization with AuNP.	[55]
GNPs functionalized with antimicrobial peptide Pediocin AcH and Listeria adhesion protein (LAP) (GNP–Pediocin–LAP)	*Listeria monocytogenes* (biofilm formation)	GNP–Pediocin–LAP showed high antibiofilm activity.	[56]
Liposomal gentamicin formulation with gallium metal (Lipo-Ga-GEN)	*P. aeruginosa* (biofilm formation); *Agrobacterium tumefaciens* A136 (AHL production).	Complete eradication of *P. aeruginosa* biofilms. Lipo-Ga-GEN prevented AHL production of *A. tumefaciens* (A136)	[57]
Liposomal Bismuth-Ethanedithiol-Loaded Tobramycin (LipoBiEDT-TOB)	*P. aeruginosa* (antimicrobial efficacy, inhibition of virulence factor production); *A. tumefaciens* strain A136 (AHL production)	Antimicrobial efficacy and reduction in virulence factor production *P. aeruginosa*. Inhibition of N-3-oxo-dodeccanoylhomoserine lactone and N-butanoylhomoserine lactone synthesis (*A. tumefaciens* strain A136).	[58]
